# The diagnostic value of long noncoding RNAs as a biomarker for in-stent restenosis in patients with coronary heart disease: A systematic review and meta-analysis of diagnostic test accuracy studies

**DOI:** 10.1097/MD.0000000000043963

**Published:** 2025-08-15

**Authors:** Shuxin Zhen, Guiping Wang, Xiaoli Li, Jing Yang, Jiaxin Yu, Yucong Wang

**Affiliations:** a Department of Internal Medicine, Tangshan Gongren Hospital, Tangshan, Hebei Province, China; b Department of Cardiology, Tangshan Gongren Hospital, Tangshan, Hebei Province, China; c Department of General Practice, Tangshan Gongren Hospital, Tangshan, Hebei Province, China; d Department of Visual Communication Design, Gengdan Institute of Beijing University of Technology, Beijing, China.

**Keywords:** analysis, coding RNA, coronary heart disease, in, long non, meta, stent restenosis

## Abstract

**Background:**

The diagnostic utility of long noncoding RNAs (lncRNAs) in detecting in-stent restenosis (ISR) among coronary heart disease (CHD) patients has drawn significant research interest. Despite this attention, however, only a limited number of lncRNAs have been translated into clinical applications. This study seeks to systematically assess the diagnostic accuracy of lncRNAs for identifying ISR in the CHD population.

**Methods:**

This meta-analysis was registered with PROSPERO (CRD420251105086).A systematic search was performed across PubMed, Web of Science, EMBASE, Wanfang, CNKI, and VIP to retrieve studies evaluating the diagnostic accuracy of lncRNAs for ISR in CHD patients, with a publication cutoff of July 2025. The diagnostic performance of lncRNAs was assessed using pooled sensitivity and specificity, positive likelihood ratio (PLR), negative likelihood ratio (NLR), summary receiver operating characteristic curve area (AUC), and diagnostic odds ratio (DOR). Statistical analyses were conducted using STATA 14.0 software.

**Results:**

A total of 7 studies involving 1020 patients were included in the analysis. The diagnostic performance of lncRNAs for identifying ISR in CHD patients was evaluated using sensitivity, specificity, PLR, NLR, DOR, and AUC. Pooled estimates revealed a sensitivity of 0.81 (95% CI: 0.75–0.86) and specificity of 0.79 (95% CI: 0.75–0.82). Corresponding PLR, NLR, and DOR were 3.8 (95% CI: 3.2–4.6), 0.24 (95% CI: 0.18–0.32), and 16 (95% CI: 11–24), respectively. The summary AUC was 0.86 (95% CI: 0.83–0.89).Subgroup analyses demonstrated significantly higher diagnostic accuracy for lncRNAs in patients with follow-up times ≤ 12 months (AUC: 0.88) compared to those with follow-up times > 12 months (AUC: 0.81). Studies with sample sizes > 100 cases exhibited superior performance (AUC: 0.89) versus those with ≤ 100 cases (AUC: 0.84). Serum-based lncRNA measurements showed a significantly higher AUC (0.89) than non-serum specimens (0.83). Diagnostic performance also varied by detection method, with RT-PCR demonstrating a higher AUC (0.87) compared to RT-qPCR (0.86). Notably, down-regulated lncRNAs exhibited better diagnostic utility (AUC: 0.86) than up-regulated lncRNAs (AUC: 0.82).

**Conclusion:**

LncRNAs demonstrate substantial diagnostic accuracy for detecting ISR in CHD patients. Nevertheless, further large-scale, prospective studies are required to validate their clinical utility and clarify their underlying molecular mechanisms.

## 1. Introduction

Coronary heart disease (CHD) arises from the progressive narrowing or occlusion of coronary arteries due to atherosclerosis, which can culminate in myocardial ischemia, hypoxia, and even necrosis.^[1,2]^ Coronary stent implantation has emerged as a cornerstone of clinical management for CHD, as it restores blood flow to ischemic myocardium and improves postoperative quality of life.^[3,4]^ Despite its efficacy, in-stent restenosis (ISR) – a pathological process characterized by neointimal hyperplasia – represents a major limitation of stent therapy.^[5,6]^ Stent deployment mechanically expands diseased vessels, inducing endothelial injury that triggers vascular smooth muscle cell proliferation, extracellular matrix deposition, and thrombus formation at the injury site.^[7,8]^ While coronary stenting remains the primary revascularization strategy for CHD, ISR contributes significantly to post-procedural morbidity and mortality, with its underlying molecular mechanisms still not fully elucidated.^[9,10]^

While drug-coated stents have substantially reduced the incidence of in-stent restenosis (ISR), this complication remains unavoidable in clinical practice.^[11,12]^ ISR occurs in 20% to 50% of cases within 3 to 6 months post-procedure, making it a persistent focus of cardiovascular research.^[13]^ Selective coronary angiography (CAG) remains the gold standard for ISR diagnosis due to its high diagnostic accuracy,^[14,15]^ but its clinical utility is constrained by invasiveness, high costs, and potential complications.^[16,17]^ Consequently, the identification of sensitive and specific biomarkers to predict ISR after coronary stenting is of critical clinical importance for mitigating complications and improving patient outcomes.

Long noncoding RNAs (lncRNAs) – nonprotein-coding RNA molecules exceeding 200 nucleotides – play pivotal roles in gene expression regulation and are implicated in diverse physiological and pathological processes.^[18]^ Numerous studies have demonstrated their involvement in cell proliferation, apoptosis, differentiation, metabolism, and immune response.^[8,19]^ During ISR pathogenesis, specific lncRNAs are upregulated and localize to injured vascular sites, where they modulate smooth muscle cell proliferation and neointimal formation – key drivers of stent-induced vascular injury.^[8,20]^ Despite growing interest, the predictive value of lncRNAs for post-stent ISR remains controversial, with conflicting results across studies.^[21–27]^ To address this knowledge gap, we conducted a systematic review and meta-analysis to synthesize current evidence on lncRNAs as diagnostic biomarkers for ISR in coronary stent recipients

## 2. Methods

This study used the Preferred Reporting Items for a Systematic Review and Meta-analysis-DTA 2018 guidelines.^[28]^ The meta-analysis was registered on the PROSPERO platform (CRD420251105086).

### 2.1. Search strategy

A systematic search was conducted across PubMed, Web of Science, EMBASE, Wanfang, CNKI, and VIP to identify studies evaluating the diagnostic accuracy of lncRNAs for ISR in CHD patients, with a publication cutoff of July 2025. The search strategy combined Medical Subject Headings (MeSH) and free words, including: (“lincRNA” OR “lncRNA” OR “Long Noncoding RNA” OR “Long Nonprotein-Coding RNA”) AND (“Coronary Disease” OR “Coronary Artery Diseases”) AND (“In-stent restenosis” OR “Restenosisin-stent” OR “ISR”) AND (“diagnosis*” OR“specificity” OR “ROC curve” OR “sensitivity”). Titles and abstracts were independently reviewed and cross-checked by 2 investigators (SXZ and YCW), and any disagreements were resolved through discussion or consultation with a third investigator (GP.W).

### 2.2. Inclusion and exclusion criteria

#### 2.2.1. Inclusion criteria

1. Patients with CHD;
2. Used invasive coronary angiography as the gold standard, defining significant restenosis as ≥ 50% diameter stenosis;
3. Study reported sensitivity, specificity, or area under the curve (AUC) values for lncRNAs in diagnosing ISR in a case-control study design;
4. Employed real-time quantitative polymerase chain reaction (RT-qPCR) or reverse transcription-polymerase chain reaction (RT-PCR) as the detection technique;
5. Provided sufficient data to construct 2 × 2 contingency tables (true positive (TP), false positive (FP), true negative (TN), false negative (FN)).

#### 2.2.2. Exclusion criteria

Studies were excluded if they:

1. Were duplicate publications;
2. Were review articles, case reports, or purely laboratory-based studies (no clinical data);
3. Did not address the diagnostic value of lncRNAs in CHD or ISR;
4. Had no accessible full-text version.

### 2.3. Data extraction

Two independent investigators (SXZ and YCW) extracted data from eligible studies using a prespecified protocol and standardized data collection form, in strict accordance with the inclusion criteria outlined above. Discrepancies in extracted data were resolved through discussion or consultation with a third investigator (GPW) to achieve consensus. The following data were systematically collected from included studies: first author, publication year, country of origin, participant ethnicity, total number of cases and controls, age distribution, sample types, lncRNA detection methods, TP, FP, FN, TN, sensitivity (SEN), and specificity (SPE).

### 2.4. Quality assessment

The risk of bias and applicability of included studies were evaluated using the quality assessment of diagnostic accuracy studies 2 (QUADAS-2) tool.^[29]^ This framework assesses bias across 4 domains – patient selection, index test, reference standard, and flow/timing – and evaluates applicability through domain-specific signaling questions. Each study was rated on 7 items: 4 addressing risk of bias and 3 addressing applicability. For each item, risk of bias was categorized as low (yes), high (no), or unclear (insufficient data to determine). All assessments were conducted independently by investigators (SXZ and YCW), with disagreements resolved through discussion.

### 2.5. Statistical analysis

Statistical analyses were conducted using Stata 14.0 software (Stata Corp LLC, College Station). The pooled estimates of diagnostic accuracy – including SEN, SPE, positive likelihood ratio (PLR), negative likelihood ratio (NLR), diagnostic odds ratio (DOR), and AUC – with 95% confidence intervals were calculated using a bivariate random-effects regression model to assess overall performance. A Spearman correlation analysis was performed to evaluate potential threshold effects. Heterogeneity across studies was assessed using Cochran *Q* statistic and the I^2^ index, with statistical significance defined as *P* < *.10* and *I*^2^ > 50% indicating substantial heterogeneity. A subgroup analysis wasconducted based on follow-up time (≤12 months vs >12 months),sample size (≤100 vs >100), sample sources (serum vs non-serum), aberrant expression (upregulation vs downregulation) and detection methods for lncRNAs (RT-qPCR vs RT-PCR). Sensitivity analyses were performed using goodness-of-fit tests and bivariate random-effects modeling to assess the stability of meta-analytic results. In the meta-analysis, the goodness-of-fit test can verify the reliability of the model when combining results from different studies, ensuring that the research data closely align with the statistical model. The bivariate random-effects model calculates the comprehensive sensitivity and specificity indicators by taking into account the differences among studies. When the 2 methods are used together, the rationality of the binary effect parameter settings can be confirmed through the model fitting degree test. Then, the sensitivity differences of different prediction indicators can be analyzed using the model, and finally, the stability and credibility of the meta-analysis results can be evaluated. Publication bias was assessed using Deeks’ funnel plot asymmetry test.

## 3. Results

### 3.1. Literature search results

**Figure 1.**
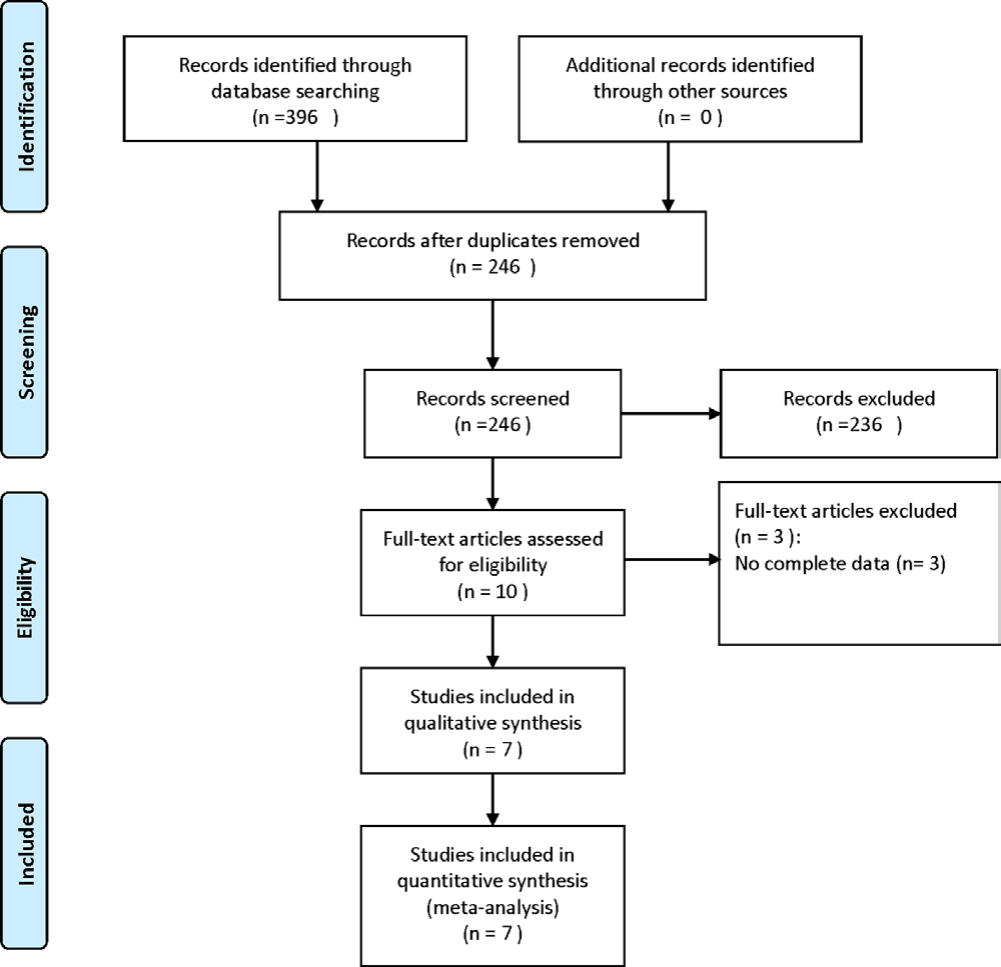
Flow diagram showing literature filtration process.

Figure 1 illustrates the literature selection process. A total of 396 articles were identified through electronic database searches. After removing 150 duplicates using Endnote, 236 articles were excluded based on title and abstract screening, leaving 10 studies for full-text review. Following detailed assessment, 7 studies^[21–27]^ were included, comprising 1020 patients.

### 3.2. Basic characteristics of the included literature

Table 1 summarizes the baseline characteristics of the included meta-analytic studies. All 7 studies^[21–27]^ were conducted in China. Low-expressed lncRNAs were categorized into 2 subtypes, whereas high-expressed lncRNAs comprised 4 distinct subtypes.

**Table 1:** Characteristics of the studies included in the meta-analysis.

First author	Year	Country	Sample (restenosis/nonrestenosis) (year)	Mean age (restenosis/nonrestenosis) (year)	Specimen	Detection method	LncRNA profiling	Follow-up time	Cut off value	SEN	SPE	AUC	Level
Zhang et al	2020	China	65/90	63.24 ± 10.12/64.25 ± 10.25	Serum	RT-qPCR	LncRNA MIR155HG	6 mo	6.55	80.00%	87.50%	0.881	High
Yin et al	2020	China	73/91	69.08 ± 7.26/68.76 ± 7.01	Serum	RT-PCR	LncRNA GAS5	6–12 mo	1.099	86.30%	73.60%	0.866	High
Zhu et al	2021	China	45/229	67.4 ± 10.7/63.4 ± 8.9	Plasma	RT-qPCR	LncRNA MALAT1	24 mo	NA	85.60%	80.10%	0.802	High
Qiu	2020	China	23/72	65.26 ± 8.15	Serum	RT-qPCR	LncRNA MALAT1	12 mo	NA	91.67%	74.65%	0.905	High
Dai	2019	China	24/46	59.35 ± 9.65/61.94 ± 9.89	PBMCs	RT-qPCR	LncRNA SENCR	6–12 mo	NA	76.70%	84.80%	0.785	Low
Wang et al	2017	China	42/220	63.0 ± 8.7/61.8 ± 6.1	Plasma	RT-qPCR	LncRNA ANRIL	36 mo	1.34	68.40%	75.00%	0.749	High
Zhao	2019	China	24/46	59.35 ± 9.65/61.94 ± 9.89	Serum	RT-PCR	LncRNA RNCR3	6–12 mo	1.204	75.00%	78.00%	0.804	Low

AUC = the area under curve, lncRNAs = long noncoding RNAs, PBMCs = peripheral blood mononuclear cells, RT-PCR = reverse transcription-polymerase chain reaction, RT-qPCR = quantitative reverse transcription polymerase chain reaction, SEN = sensitivity, SPE = specificity.

### 3.3. Assessment of study quality

Table 2 presents the quality assessment results using the QUADAS-2 tool.

**Table 2:** Methodological quality evaluation of including literature.

Study	Risk of bias				Applicability		
	Patient selection	Index test	Reference standard	Flow and timing	Patient selection	Index test	Reference standard
Zhang et al 2020	U	L	U	L	L	L	L
Yin et al 2020	U	L	U	L	L	L	L
Zhu et al 2021	U	U	U	L	L	L	L
Qiu 2020	U	L	U	L	L	L	L
Dai 2019	U	L	L	L	L	L	L
Wang et al 2017	L	L	U	L	L	L	L
Zhao 2019	U	L	U	L	L	L	L

H = high risk of bias, L = low risk of bias, U = unclear risk of bias.

### 3.4. Threshold effect analysis and heterogeneity test

No significant threshold effect was observed, as evidenced by a Spearman correlation coefficient of 0.03 (*P* = .*170*). The *I*^2^ values for SEN and SPE were 32.90% (*P* = .18 > 0.1) and 36.17% (P = .15 > 0.1), respectively, indicating low heterogeneity.

### 3.5. Results of the pooled analysis

**Figure 2.**
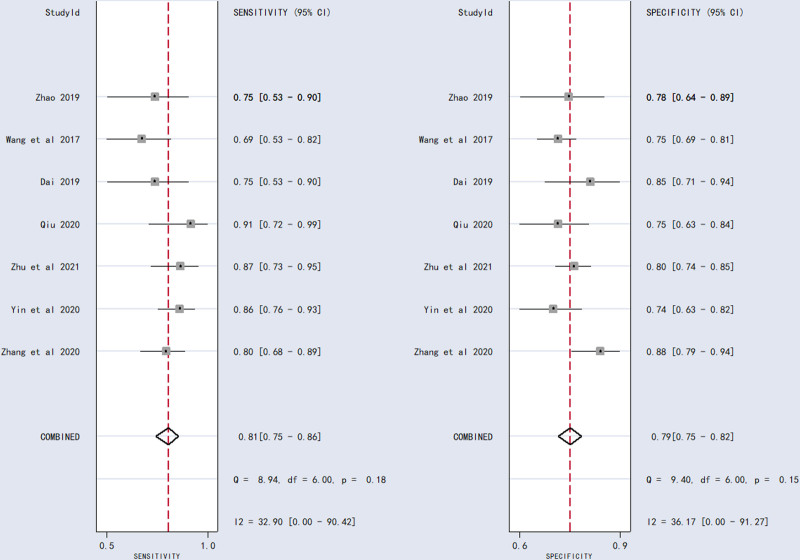
Forest plots of summary sensitivities and specificity.

**Figure 3.**
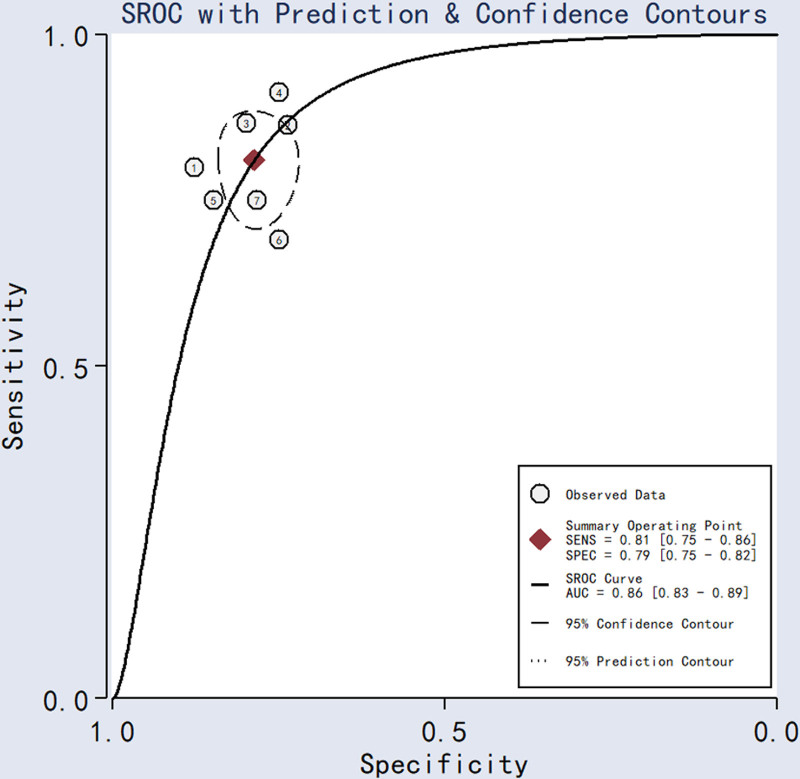
Summary ROC curves for lncRNAs in the diagnosis of ISR. ISR = in-stent restenosis, lncRNAs = long noncoding RNAs.

SEN, SPE, PLR, NLR, DOR, and AUC were calculated based on the values of TP, TN, FP, and FN extracted from the included studies. Meta-analytic pooled estimates with random-effects weighting yielded: SEN = 0.81 (95% CI: 0.75–0.86), SPE = 0.79 (95% CI: 0.75–0.82), PLR = 3.8 (95% CI: 3.2–4.6), NLR = 0.24 (95% CI: 0.18–0.32), DOR = 16 (95% CI: 11–24), and AUC = 0.86 (95% CI: 0.83–0.89). Forest plots and SROC curves for the 7 studies^[21–27]^ are shown in Figures 2 and 3.

### 3.6. Subgroup analysis

Subgroup analyses were performed to investigate sources of heterogeneity and evaluate diagnostic performance across predefined characteristics, with results summarized in Table 3. For follow-up duration, lncRNAs demonstrated significantly higher diagnostic accuracy in studies with follow-up times ≤ 12 months (AUC: 0.88) compared to those with follow-up times > 12 months (AUC: 0.81). Studies with sample sizes > 100 cases exhibited superior diagnostic performance (AUC: 0.89) versus those with ≤ 100 cases (AUC: 0.84).

**Table 3:** Summary estimates of diagnostic criteria and their 95% confidence intervals.

Subgroup	N	SEN (95% CI)	SPE (95% CI)	DOR (95% CI)	AUC
Follow-up time					
≤12 mo	5	0.82 (0.76–0.87)	0.80 (0.75–0.84)	19.53 (12.28–31.07)	0.88 (0.76–0.92)
>12 mo	2	0.78 (0.68–0.86)	0.78 (0.73–0.81)	12.76 (3.38–48.14)	0.81 (0.74–0.89)
Sample size					
≤100	3	0.76 (0.66–0.85)	0.76 (0.71–0.81)	12.61 (5.03–31.62)	0.84 (0.78–0.90)
>100	4	0.83 (0.77–0.88)	0.80 (0.76–0.84)	20.53 (12.97–32.50)	0.89 (0.84–0.97)
Specimen					
Serum	4	0.83 (0.77–0.88)	0.79 (0.74–0.83)	20.05 (12.14–33.11)	0.89 (0.83–0.96)
Non-serum	3	0.78 (0.69–0.85)	0.78 (0.74–0.82)	13.51 (5.52–33.15)	0.83 (0.72–0.90)
Altered LncRNA					
Upregulation	5	0.82 (0.77–0.87)	0.78 (0.75–0.81)	17.79 (9.54–33.17)	0.86 (0.82–0.96)
Downregulation	2	0.75 (0.60–0.86)	0.82 (0.72–0.89)	13.28 (5.72–30.82)	0.82 (0.74–0.92)
Method					
RT-qPCR	5	0.80 (0.74–0.85)	0.79 (0.76–0.82)	17.77 (9.04–34.94)	0.86 (0.78–0.92)
RT-PCR	2	0.84 (0.75–0.90)	0.75 (0.67–0.82)	14.97 (7.69–29.15)	0.87 (0.80–0.92)
Overall	–	0.81 (0.75–0.86)	0.79 (0.75–0.82)	16 (11–24)	0.86 (0.83–0.89)

AUC = the area under curve, Cl = confidence intervals, DOR = diagnostic odds ratio, N = number, RT-PCR = reverse transcription-polymerase chain reaction, RT-qPCR = quantitative reverse transcription polymerase chain reaction, SEN = sensitivity, SPE = specificity.

Regarding sample type, serum-derived lncRNAs showed a significantly higher AUC (0.89) than non-serum specimens (0.83). By detection method, RT-PCR yielded better diagnostic performance (AUC: 0.87) compared to real-time quantitative PCR (RT-qPCR, AUC: 0.86). Notably, down-regulated lncRNAs demonstrated superior diagnostic utility (AUC: 0.86) versus up-regulated lncRNAs (AUC: 0.82).

### 3.7. Analysis of sensitivity

**Figure 4.**
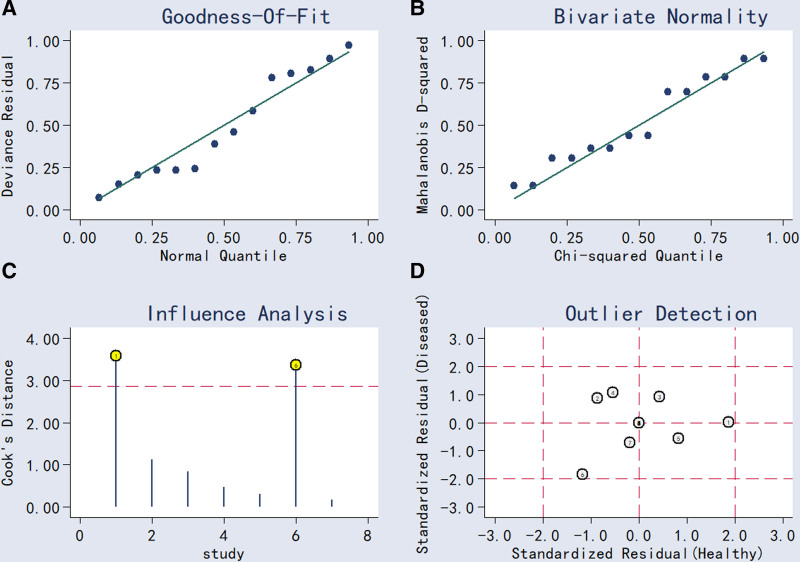
Sensitivity analysis:graphicai depiction of goodness-of-fit and bivariate normality analysis (A and B) influence and outlier detection analysis (C and D) respectively.

Sensitivity analyses were performed using goodness-of-fit tests and bivariate random-effects modeling to assess the stability of meta-analytic results. The random-effects model was confirmed as appropriate for pooled estimation, as demonstrated by fit statistics (see Fig. 4A and B). Impact analysis and outlier detection identified 2 studies with potential influence (Fig. 4C and D). Excluding these studies resulted in a marginal increase in sensitivity (SEN: 0.81 → 0.84) and a slight decrease in specificity (SPE: 0.79 → 0.78). The PLR remained unchanged, while the NLR decreased from 0.24 to 0.20. The DOR increased from 16 to 19, and AUC rose from 0.86 to 0.88. Collectively, these sensitivity analyses indicate that the core findings are robust and not driven by individual studies (Fig. 4).

### 3.8. Publication bias

**Figure 5.**
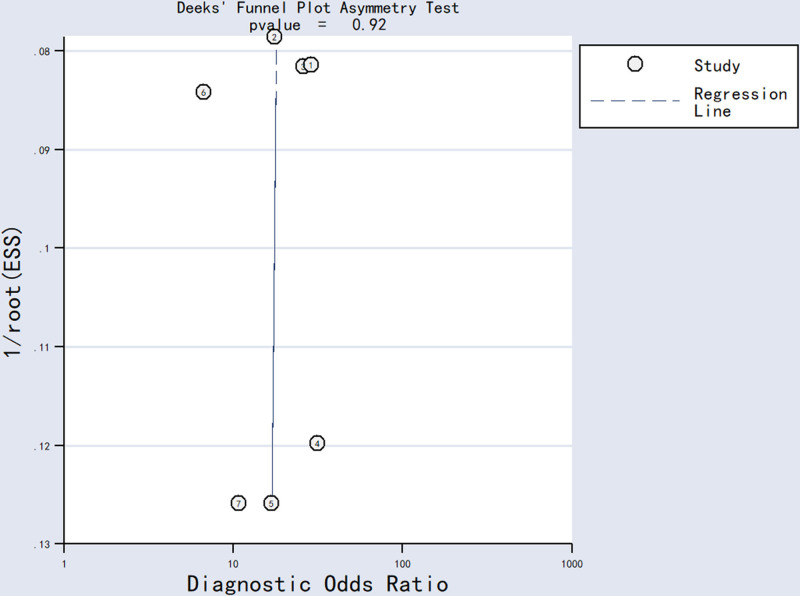
Deeks’ funnel plot asymmetry test for the assessment of tential publication bias.

Deek funnel chart asymmetry test was used to evaluate publication bias in the diagnostic accuracy meta-analysis. No evidence of publication bias was detected (*P* = .92) (Fig. 5).

## 4. Discussion

Coronary artery stent implantation remains a cornerstone of clinical therapy for CHD. However, ISR ollowing stenting represents a major challenge that compromises long-term treatment efficacy and patient outcomes.^[30]^ While anticoagulant therapies and drug-eluting stents have reduced ISR incidence,^[31,32]^ this complication continues to hinder the broader success of coronary stenting and threatens patient safety. Proactive prediction of ISR risk and implementation of early interventional strategies – either pre- or post-procedurally – are therefore critical for minimizing ISR burden and improving clinical outcomes.

LncRNAs exhibit frequent dysregulation in diverse diseases, including malignant tumors and cardiovascular and neurological disorders, positioning them as promising diagnostic biomarkers and therapeutic targets.^[33–35]^ Growing evidence highlights their role in atherosclerosis, with recent studies identifying abnormal plasma levels of 5 lncRNAs (upregulated) and 57 lncRNAs (downregulated) in CHD patients.^[36]^ Mechanistically, lncRNAs influence atherosclerosis progression by modulating vascular smooth muscle cell proliferation and apoptosis,^[37–39]^ while also contributing to genetic susceptibility to CHD – findings that underpin their potential for early screening.^[40,41]^

This meta-analysis synthesized data on 6 dysregulated lncRNAs (2 upregulated, 4 downregulated), demonstrating their combined ability to predict ISR in CHD patients with AUC of 0.86. Pooled sensitivity and specificity were 0.81 and 0.79, respectively, indicating robust diagnostic accuracy. Collectively, these findings establish lncRNAs as a valuable biomarker for ISR diagnosis in CHD, with translational potential for clinical use as an adjunctive tool in ISR evaluation.

Among the 6 lncRNAs analyzed, MALAT1 demonstrated the strongest diagnostic performance for ISR in CHD patients, with an AUC of 0.905. Widely studied in cardiovascular disease, MALAT1 is highly expressed in the peripheral vasculature, where it regulates coronary artery smooth muscle cell proliferation and migration.^[42]^ Endothelial injury induced by inflammation is a central driver of stent restenosis, and MALAT1 – abundantly expressed in endothelial cells – has been linked to endothelial dysfunction, inflammatory signaling, and angiogenesis.

Notably, ideal biomarkers necessitate measurement in minimally invasive matrices. Subgroup analysis revealed significantly higher diagnostic utility for serum-derived lncRNAs compared to non-serum specimens. Additionally, follow-up duration impacted diagnostic accuracy: studies with ≤ 12 months of follow-up exhibited superior performance (AUC = 0.88) versus longer intervals, highlighting the role of temporal dynamics in ISR detection.

This meta-analysis has several notable limitations. First, the small number of included studies (n = 7) may compromise the robustness of the findings and limit statistical power. Second, all included research was conducted in Chinese populations, which introduces geographic and ethnic bias and restricts generalizability to diverse global cohorts. Third, individual lncRNAs exhibit distinct molecular mechanisms in ISR pathogenesis, yet most studies evaluated single lncRNAs rather than combinatorial panels, potentially overlooking synergistic diagnostic effects. Additionally, the reliance on cross-sectional data hinders assessment of lncRNA dynamics over time. Collectively, these limitations – including sample size constraints, lack of ethnic diversity, and methodological heterogeneity – warrant cautious interpretation of the results. Prospective research should prioritize large-scale, multicenter studies with diverse populations to enhance generalizability. Incorporating longitudinal follow-up and combinatorial lncRNA panels will help clarify their temporal utility and additive diagnostic value. Furthermore, mechanistic studies exploring lncRNA-protein interactions in ISR pathogenesis are needed to translate these findings into clinical applications.

## 5. Conclusions

In summary, lncRNAs represent promising biomarkers for predicting ISR in CHD patients. Although lncRNA-based testing holds clinical utility for identifying ISR, results should be interpreted alongside clinical context and other diagnostic modalities.

## Author contributions

**Conceptualization:** Shuxin Zhen, Yucong Wang.

**Data curation:** Guiping Wang, Yucong Wang.

**Formal analysis:** Shuxin Zhen, Guiping Wang.

**Funding acquisition:** Shuxin Zhen.

**Investigation:** Xiaoli Li, Yucong Wang.

**Methodology:** Guiping Wang, Xiaoli Li, Yucong Wang.

**Project administration:** Shuxin Zhen.

**Resources:** Xiaoli Li, Jing Yang.

**Software:** Xiaoli Li.

**Supervision:** Shuxin Zhen.

**Validation:** Jing Yang, Jiaxin Yu.

**Visualization:** Jing Yang, Jiaxin Yu.

**Writing – original draft:** Shuxin Zhen.

**Writing – review & editing:** Shuxin Zhen.
